# Supplementary material for: In Silico and Experimental Investigation of the Biological Potential of Some Recently Developed Carprofen Derivatives
Source: Molecules. 2022 Apr 23;27(9):2722. doi: 10.3390/molecules27092722 (PMC9101252; doi:10.3390/molecules27092722)
Supplement: Supplementary file 1 [file molecules-27-02722-s001.zip › molecules-1693817-supplementary.pdf]

molecules

Supplementary Materials

In Silico and Experimental Investigation of the Biological Potential of Some Recently Developed Carprofen Derivatives

Florea Dumitrascu [1,†], Ana-Maria Udrea [2,3,†], Mino R. Caira [4,*], Diana Camelia Nuta [5,*], Carmen Limban [5], Mariana Carmen Chifiriuc [6,7,8], Marcela Popa [6], Coralia Bleotu [2,6,9], Anamaria Hanganu [1], Denisa Dumitrescu [10] and Speranta Avram [11]

[1] Center for Organic Chemistry "C.D. Nenitzescu", Romanian Academy, Spl. Independentei 202B, 060023 Bucharest, Romania; fdumitra@yahoo.com (F.D.), ana.hanganu@cco.ro (A.H.)
[2] Research Institute of the University of Bucharest—ICUB, University of Bucharest, 91-95 Splaiul Independentei, 050095 Bucharest, Romania; ana.udrea@inflpr.ro (A.-M.U.); cbleotu@yahoo.com (C.B.)
[3] National Institute of Laser, Plasma and Radiation Physics, 409 Atomistilor Str., 077125 Magurele, Romania
[4] Department of Chemistry, University of Cape Town, Rondebosch 7701, South Africa
[5] Department of Pharmaceutical Chemistry, Faculty of Pharmacy, "Carol Davila" University of Medicine and Pharmacy, 6 TraianVuia Street, 020956 Bucharest, Romania; carmen.limban@umfcd.ro
[6] Department of Microbiology, Faculty of Biology, University of Bucharest, 1-3 Aleea Portocalelor Str., District 5, 060101 Bucharest, Romania; carmen.chifiriuc@bio.unibuc.ro (M.C.C.); marcela.popa@bio.unibuc.ro (M.P.)
[7] Romanian Academy of Scientists, 54 Spl. Independenței Str., District 5, 50085 Bucharest, Romania
[8] The Romanian Academy, 25, Calea Victoriei, Sector 1, District 1, 010071 Bucharest, Romania
[9] Department of Cellular and Molecular Pathology, Stefan S. Nicolau Institute of Virology, 030304 Bucharest, Romania
[10] Faculty of Pharmacy, Ovidius University Constanta, Str. Cpt. Av. Al. Serbanescu, Campus Corp C, 900470 Constanta, Romania; denisa.dumitrescu2014@gmail.com
[11] Department of Anatomy, Animal Physiology, and Biophysics, Faculty of Biology, University of Bucharest, 36-46 M. Kogălniceanu Boulevard, 050107 Bucharest, Romania; speranta.avram@gmail.com
* Correspondence: mino.caira@uct.ac.za (M.R.C.); diana.nuta@umfcd.ro (D.C.N.); Tel.: +40-213-180-739 (D.C.N.)
† These authors equally contribute to this work.

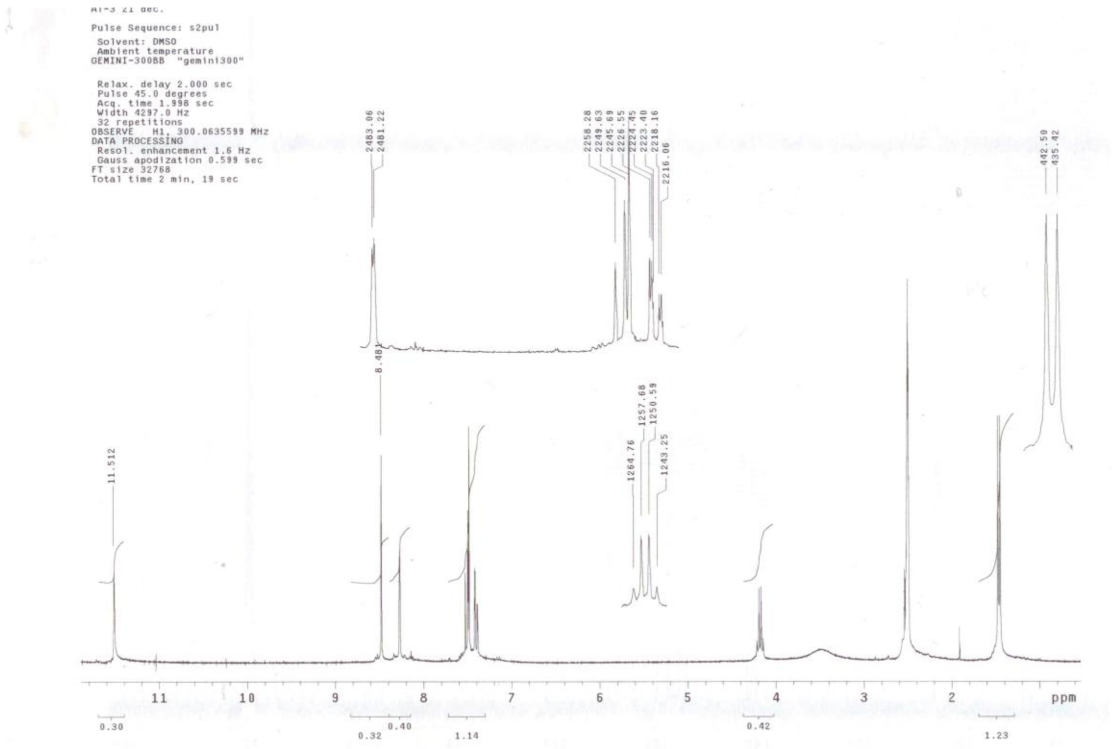

Figure S1a. The ^{1}H-NMR spectrum of 2-(3-Bromo-6-chloro-9H-carbazol-2-yl)propanoic acid (**2**).

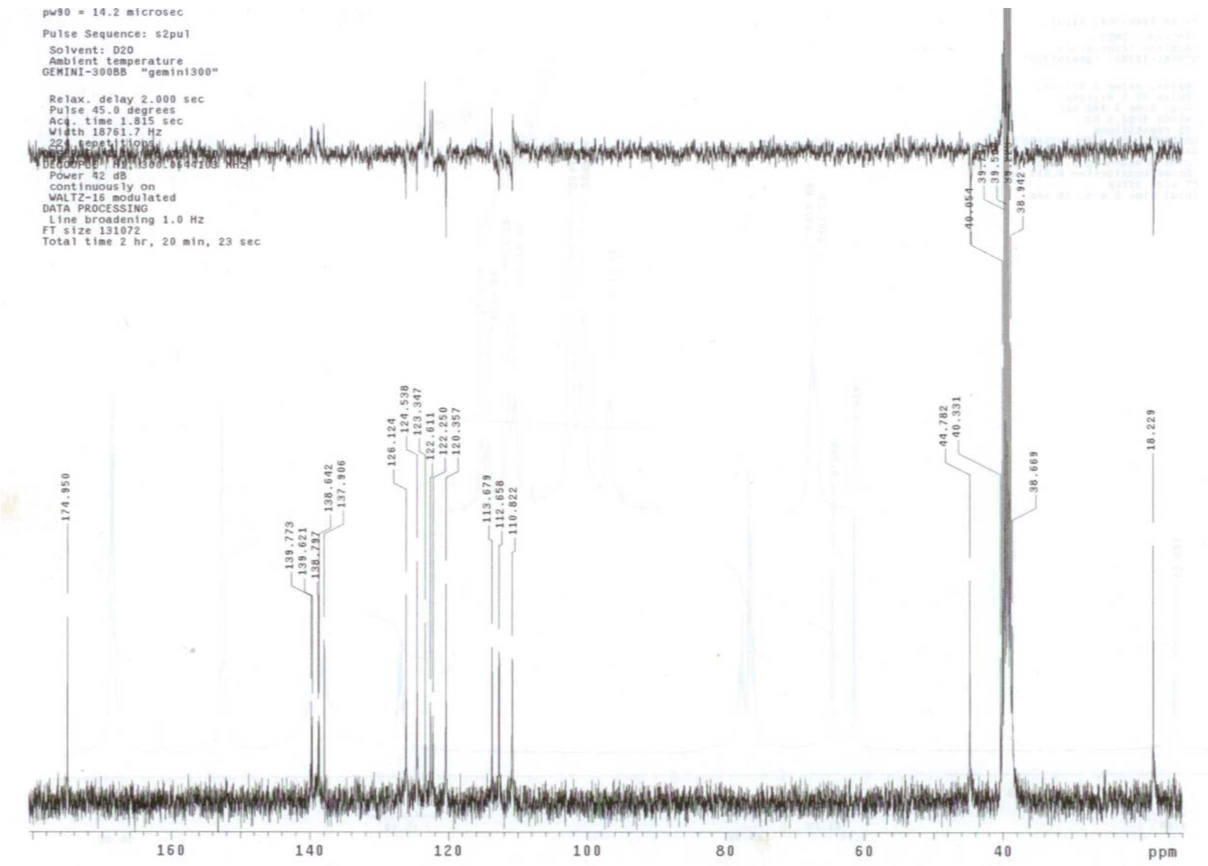

Figure S1b. The ^{13}C-NMR spectrum of 2-(3-Bromo-6-chloro-9H-carbazol-2-yl)propanoic acid (**2**).

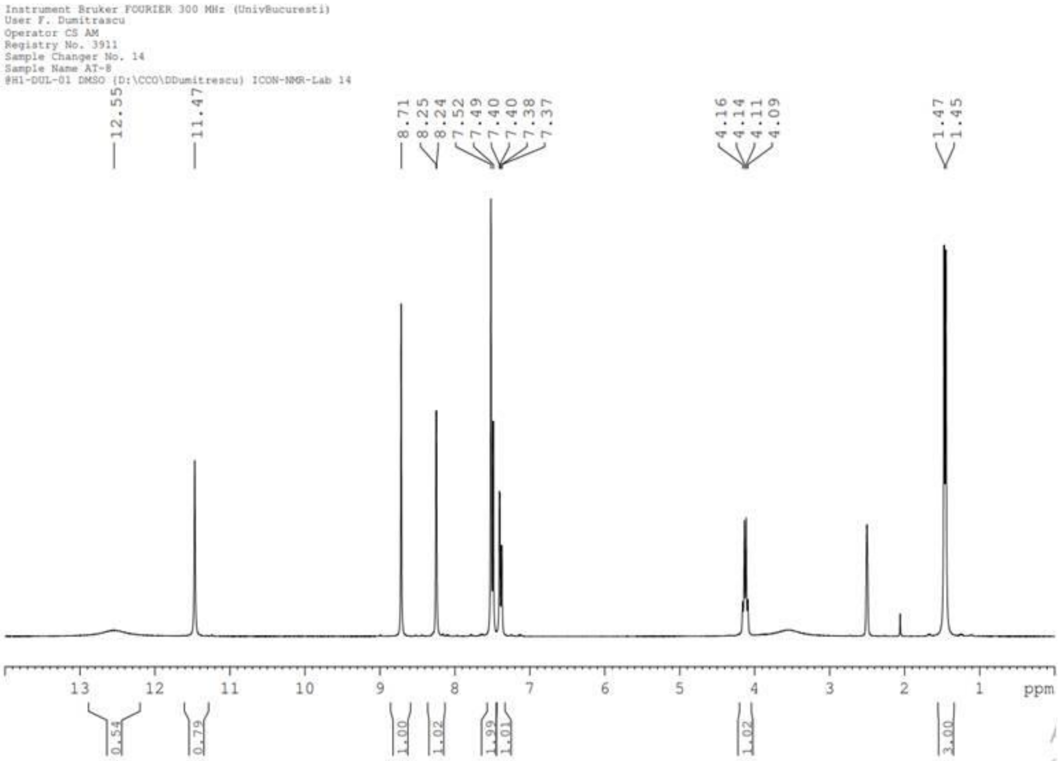

Figure S2a. The ^{1}H-NMR spectrum of 2-(3-Iodo-6-chloro-9H-carbazol-2-yl)propanoic acid (**3**).

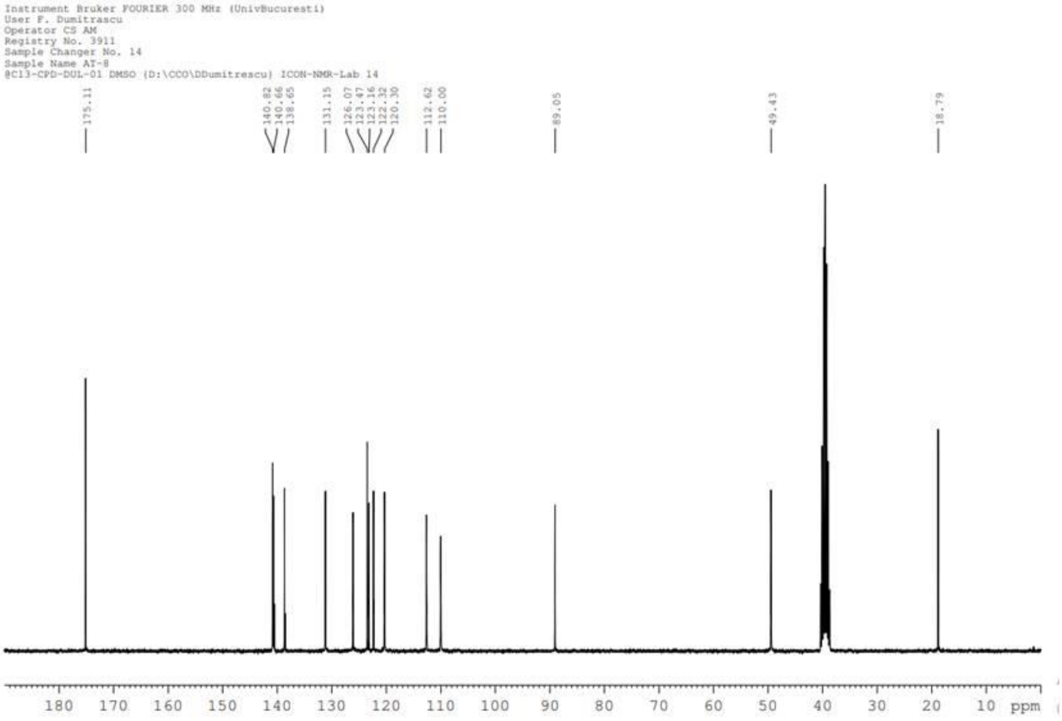

Figure S2b. The ^{13}C-NMR spectrum of 2-(3-Iodo-6-chloro-9H-carbazol-2-yl)propanoic acid (**3**).

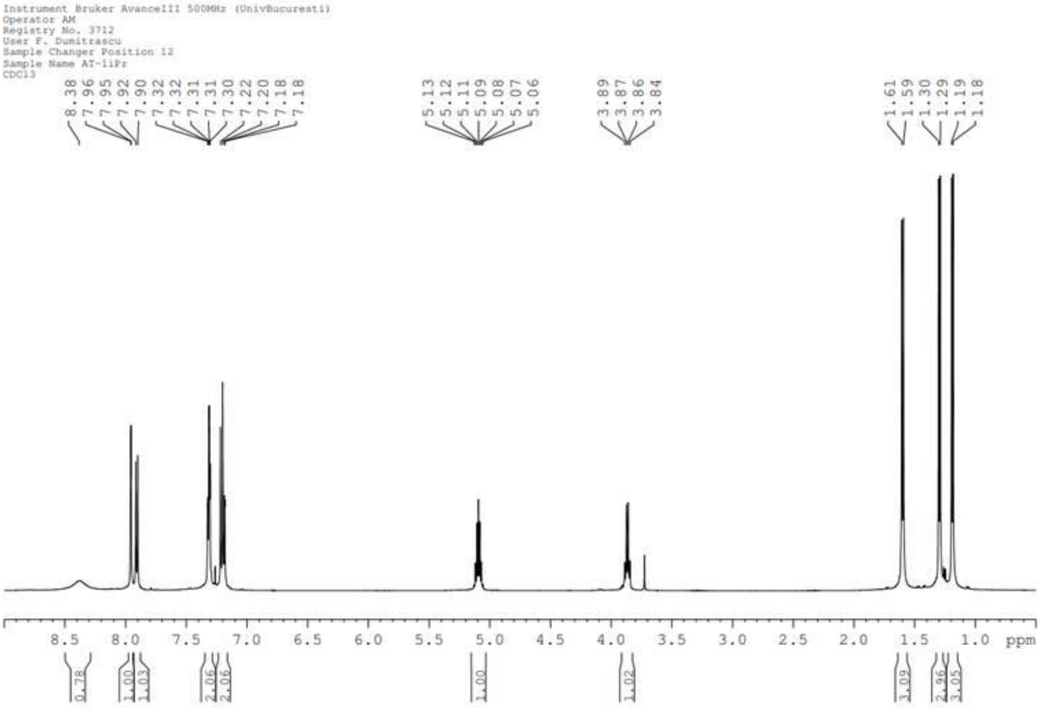

Figure S3a. The ^{1}H-NMR spectrum of Isopropyl 2-(6-chloro-9H-carbazol-2-yl)propanoate (**4**).

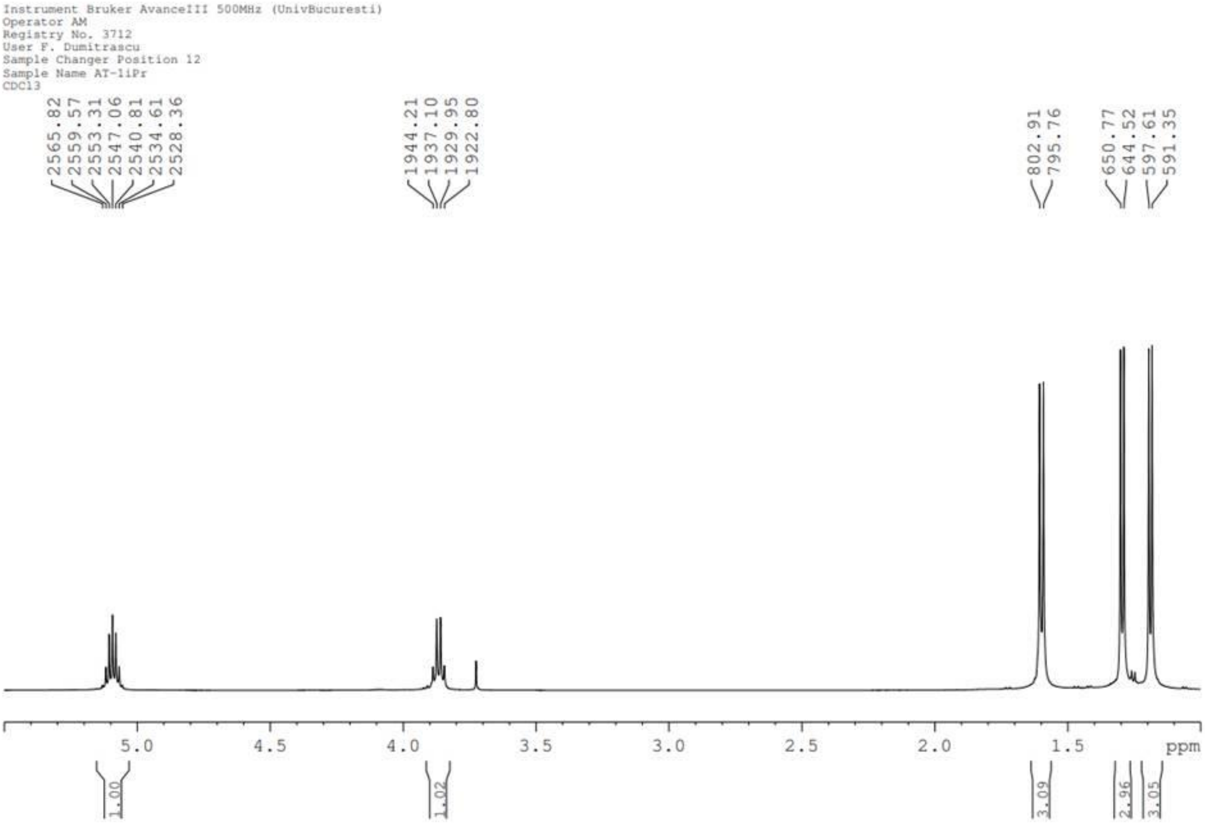

Figure S3b. The ^{1}H-NMR spectrum (details) of Isopropyl 2-(6-chloro-9H-carbazol-2-yl)propanoate (**4**).

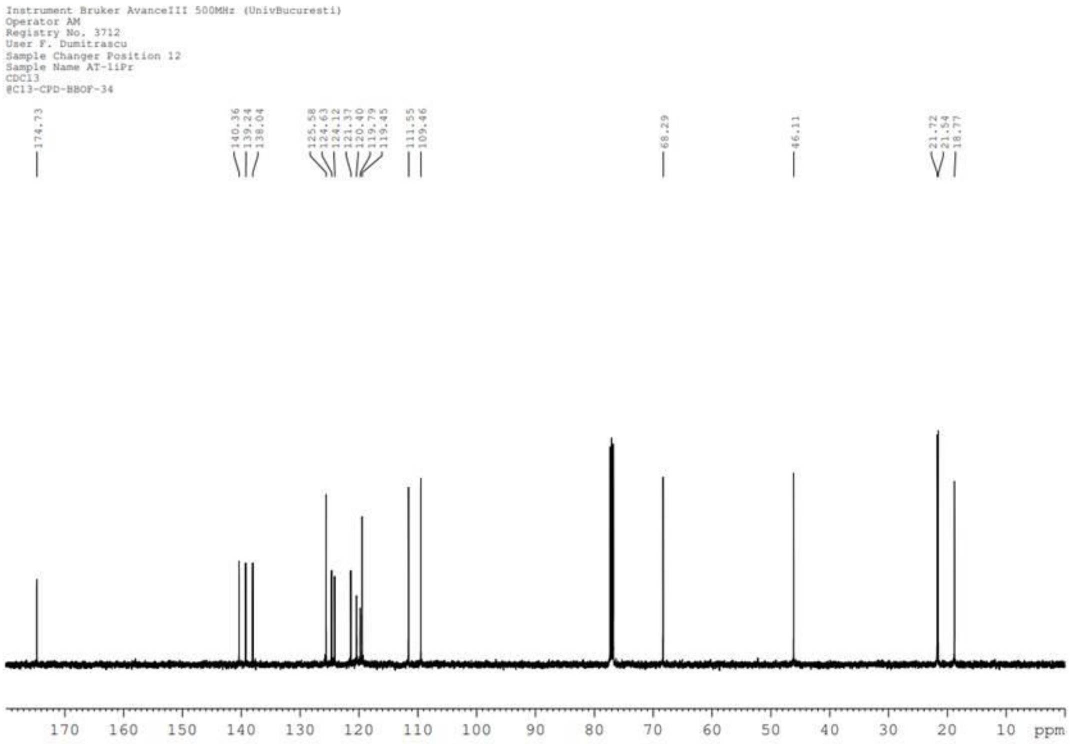

Figure S3c. The ^{13}C-NMR spectrum of Isopropyl 2-(6-chloro-9H-carbazol-2-yl)propanoate (**4**).

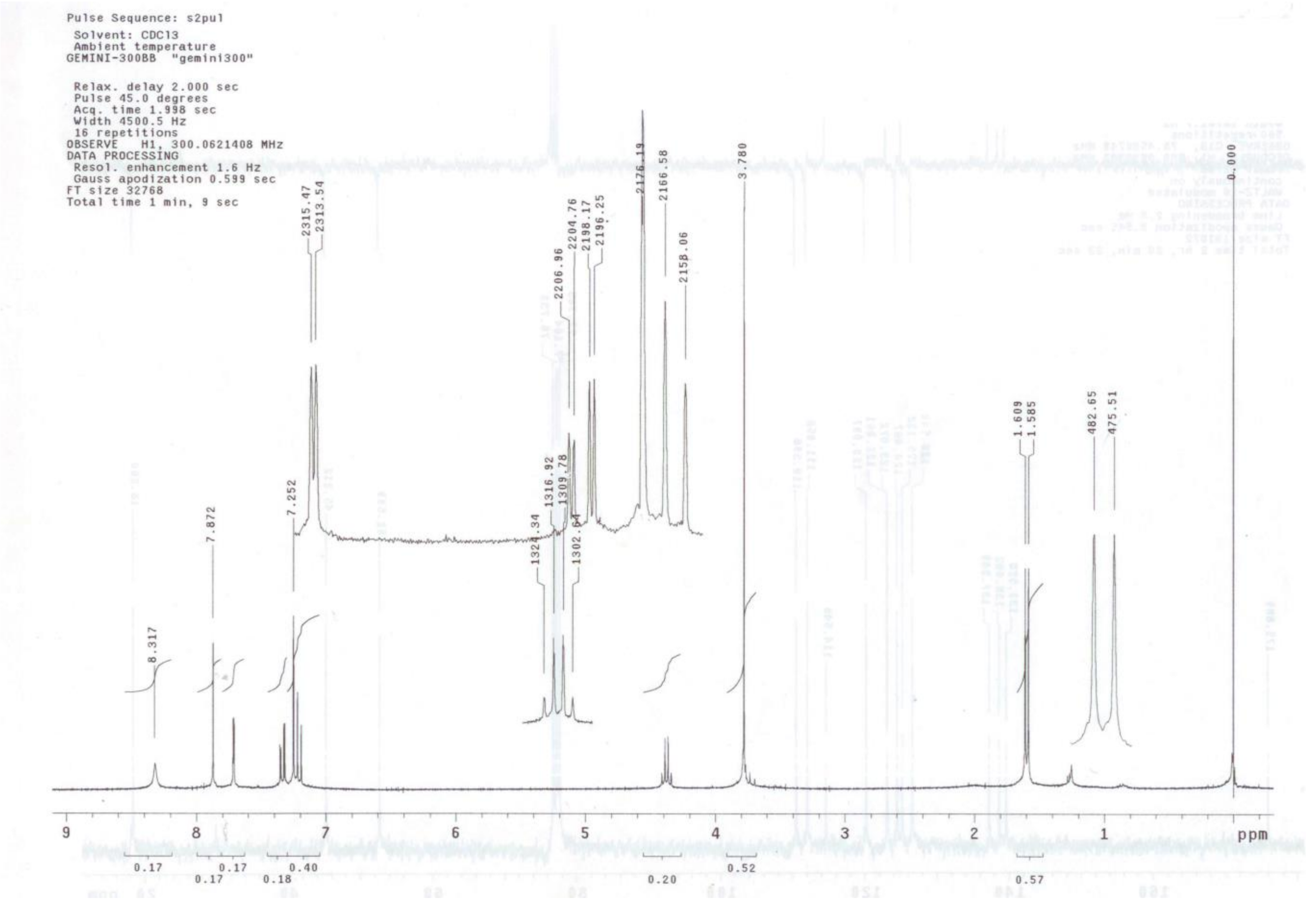

Figure S4a. The ^{1}H-NMR spectrum of Methyl 2-(3-Bromo-6-chloro-9H-carbazol-2-yl)propanoate (**5**).

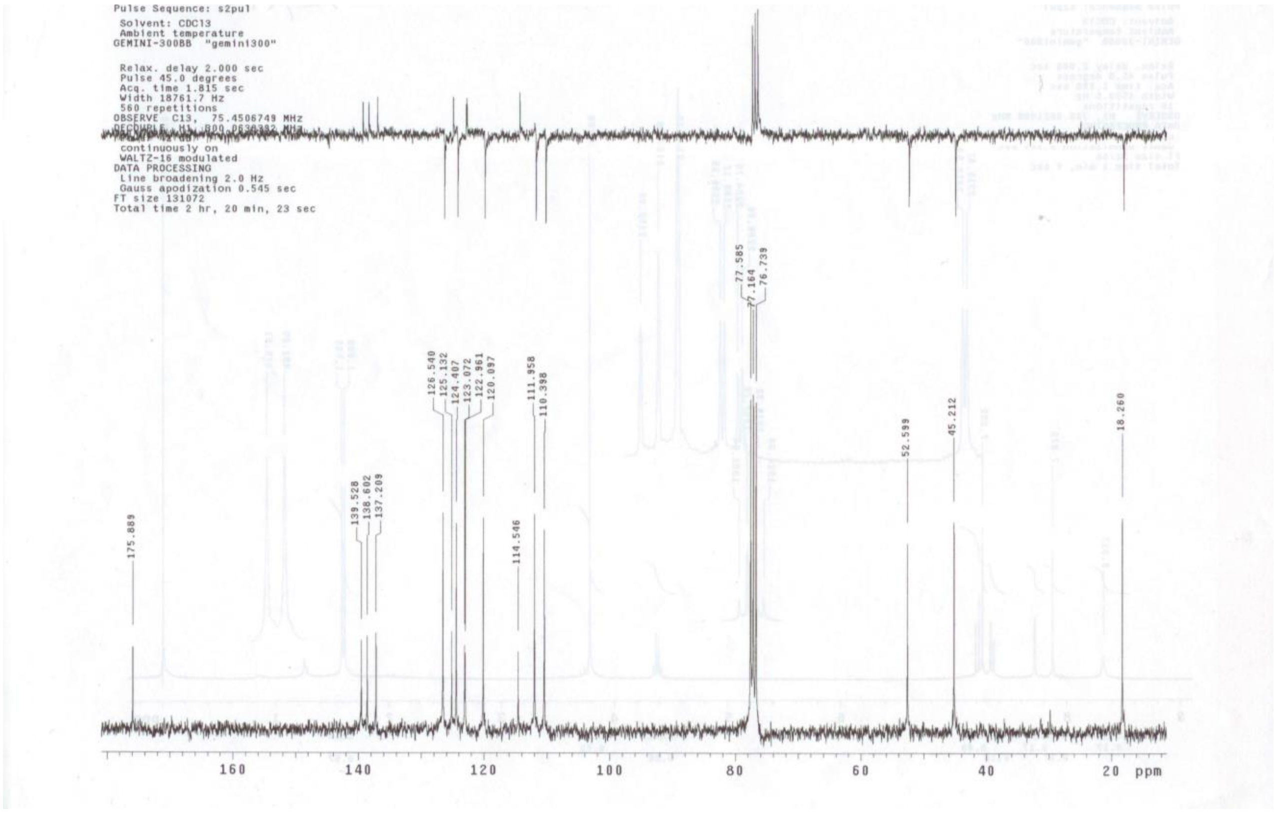

Figure S4b. The ^{13}C-NMR spectrum of Methyl 2-(3-Bromo-6-chloro-9H-carbazol-2-yl)propanoate (**5**).

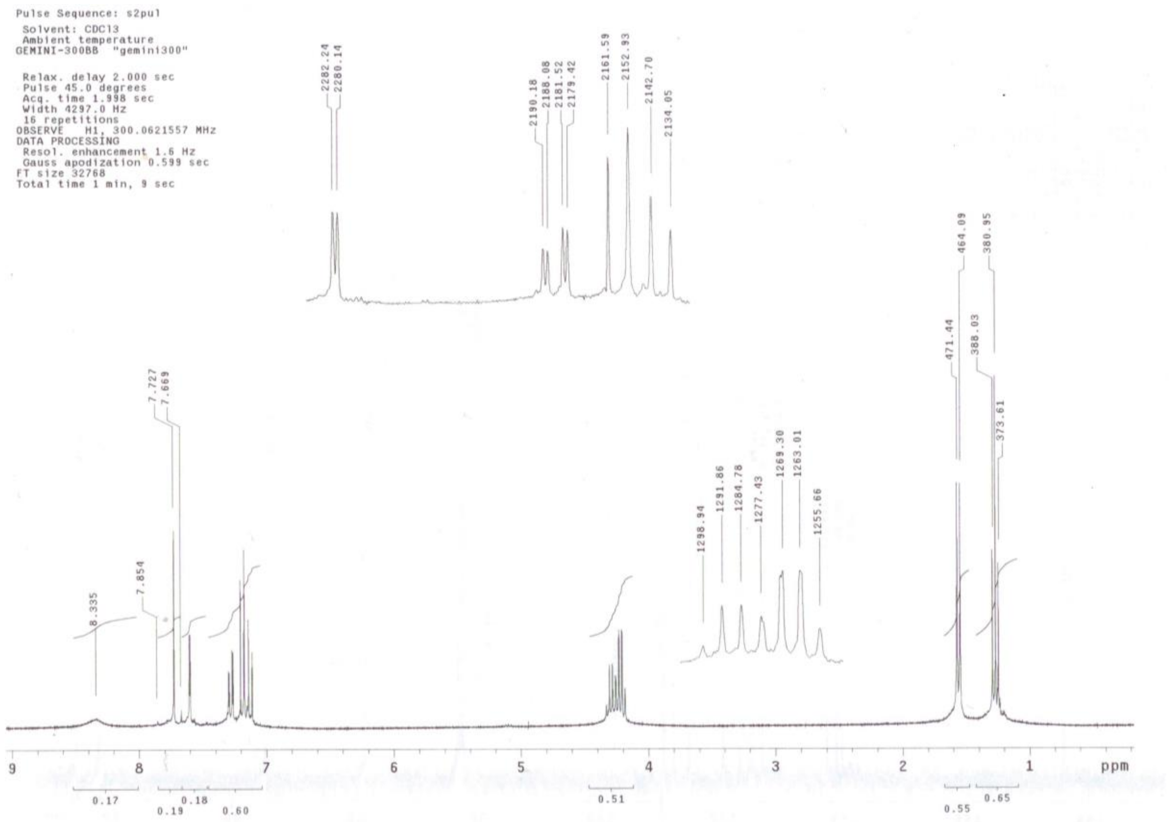

Figure S5a. The ^{1}H-NMR spectrum of Ethyl 2-(3-Bromo-6-chloro-9H-carbazol-2-yl)propanoate (**6**).

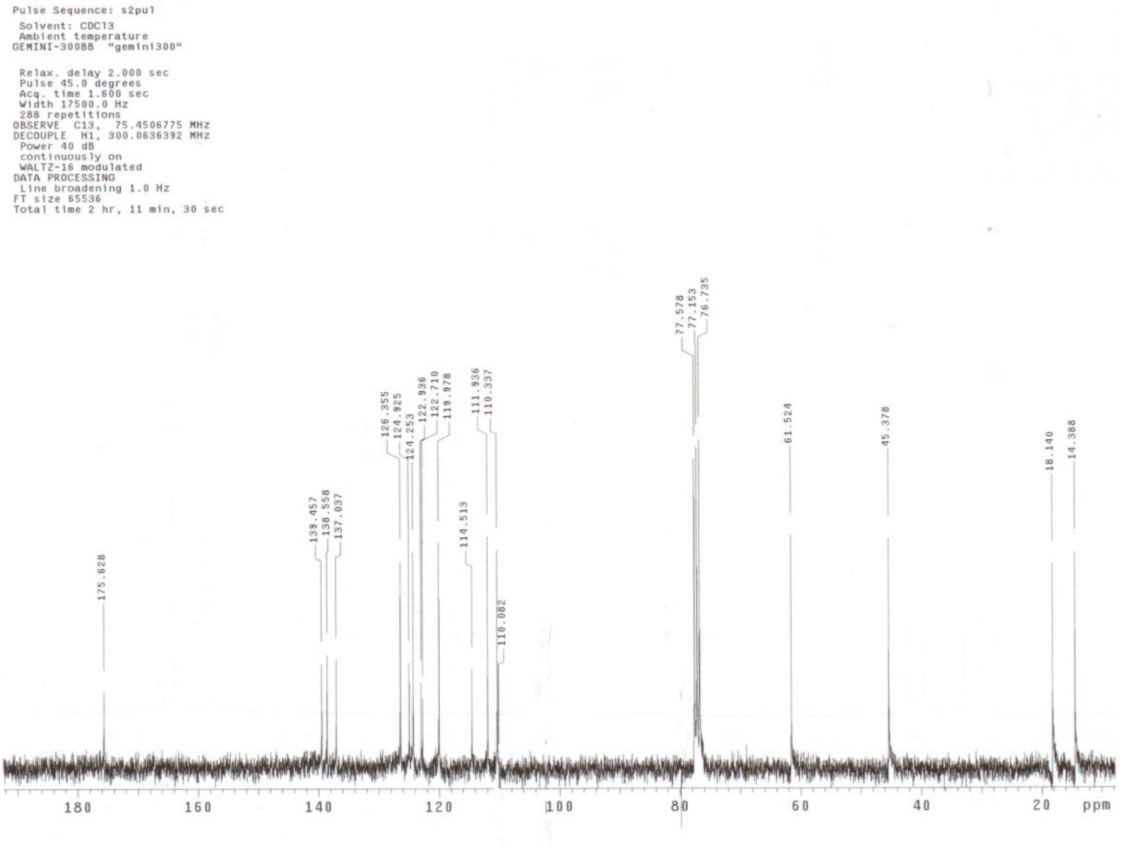

Figure S5b. The ^{13}C-NMR spectrum of Ethyl 2-(3-Bromo-6-chloro-9H-carbazol-2-yl)propanoate (**6**).

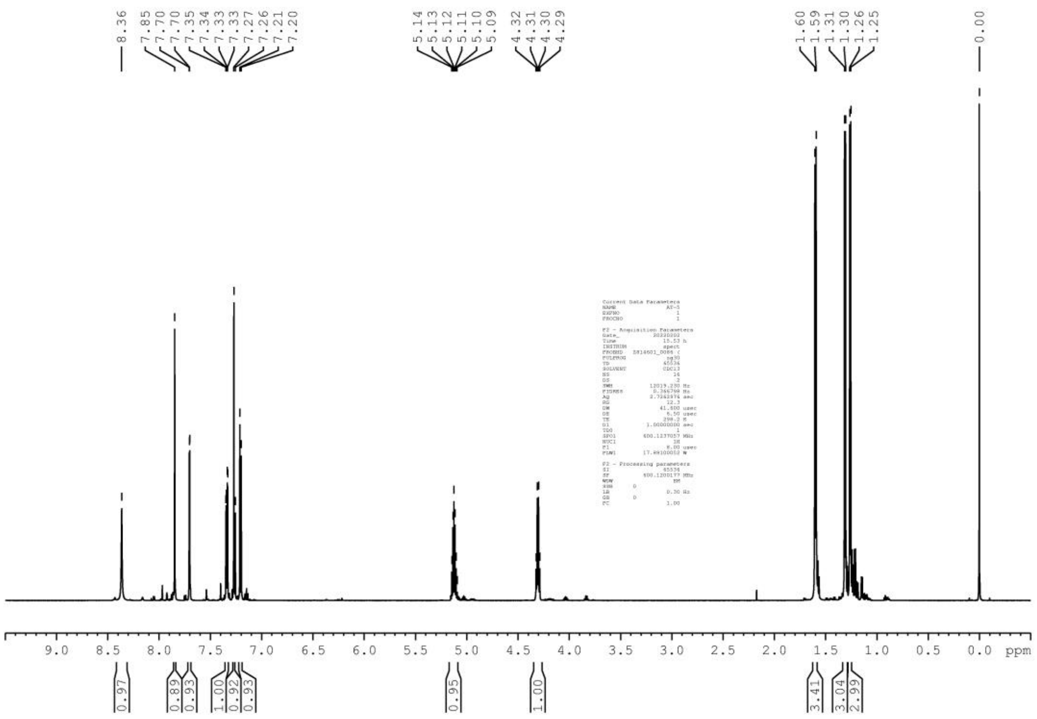

Figure S6a. The ^{1}H-NMR spectrum of Isopropyl 2-(3-Bromo-6-chloro-9H-carbazol-2-yl)propanoate (**7**).

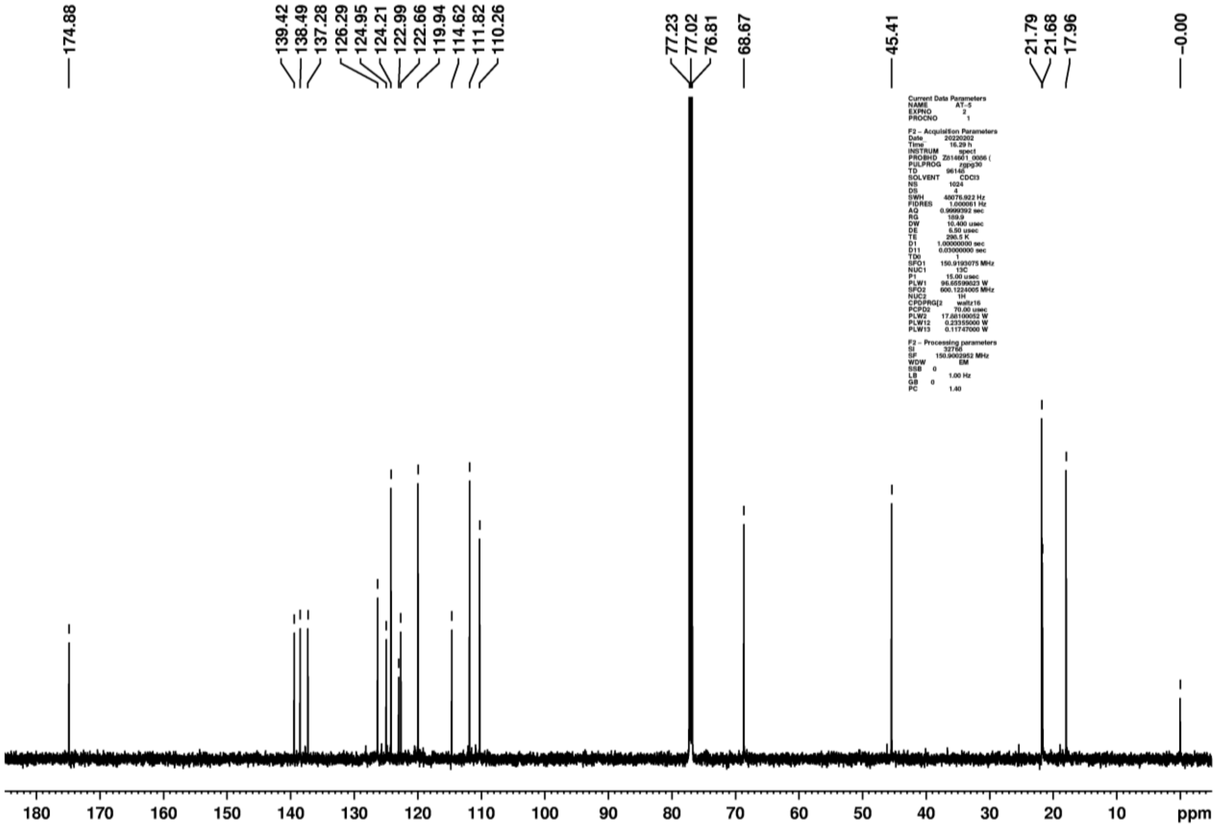

Figure S6b. The ^{13}C-NMR spectrum of Isopropyl 2-(3-Bromo-6-chloro-9H-carbazol-2-yl)propanoate (**7**).

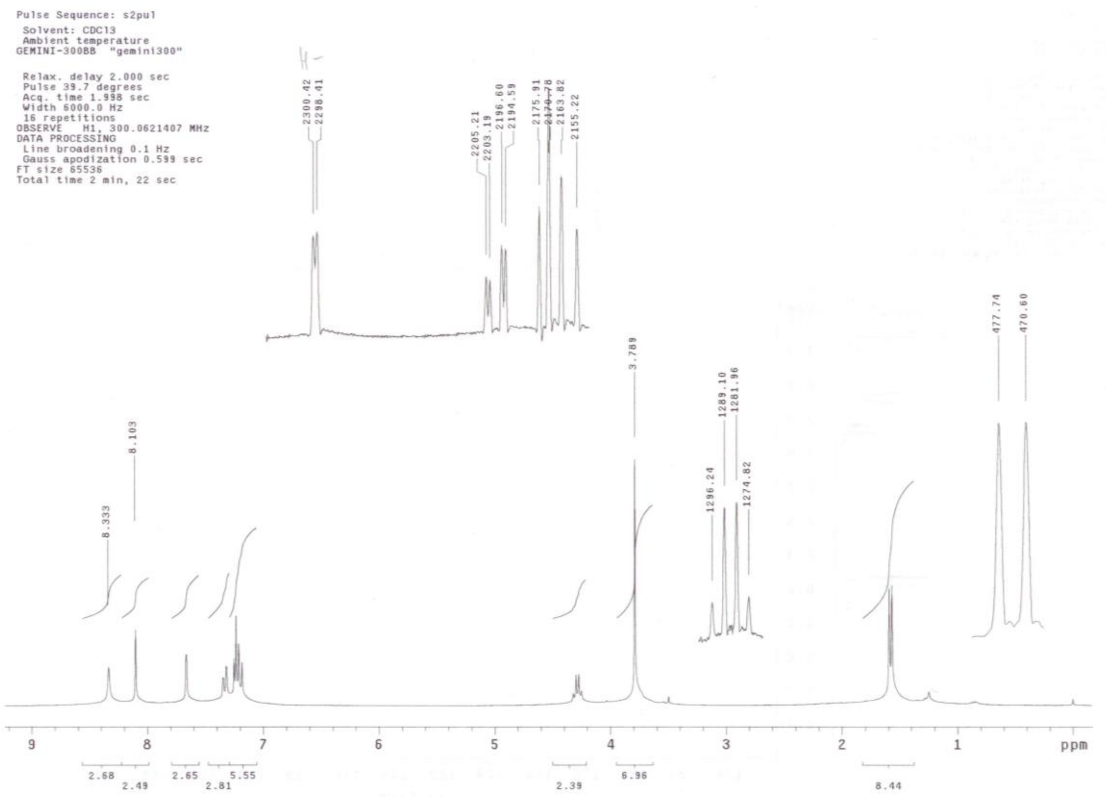

Figure S7a. The ^{1}H-NMR spectrum of Methyl 2-(3-iodo-6-chloro-9H-carbazol-2-yl)propanoate (**8**).

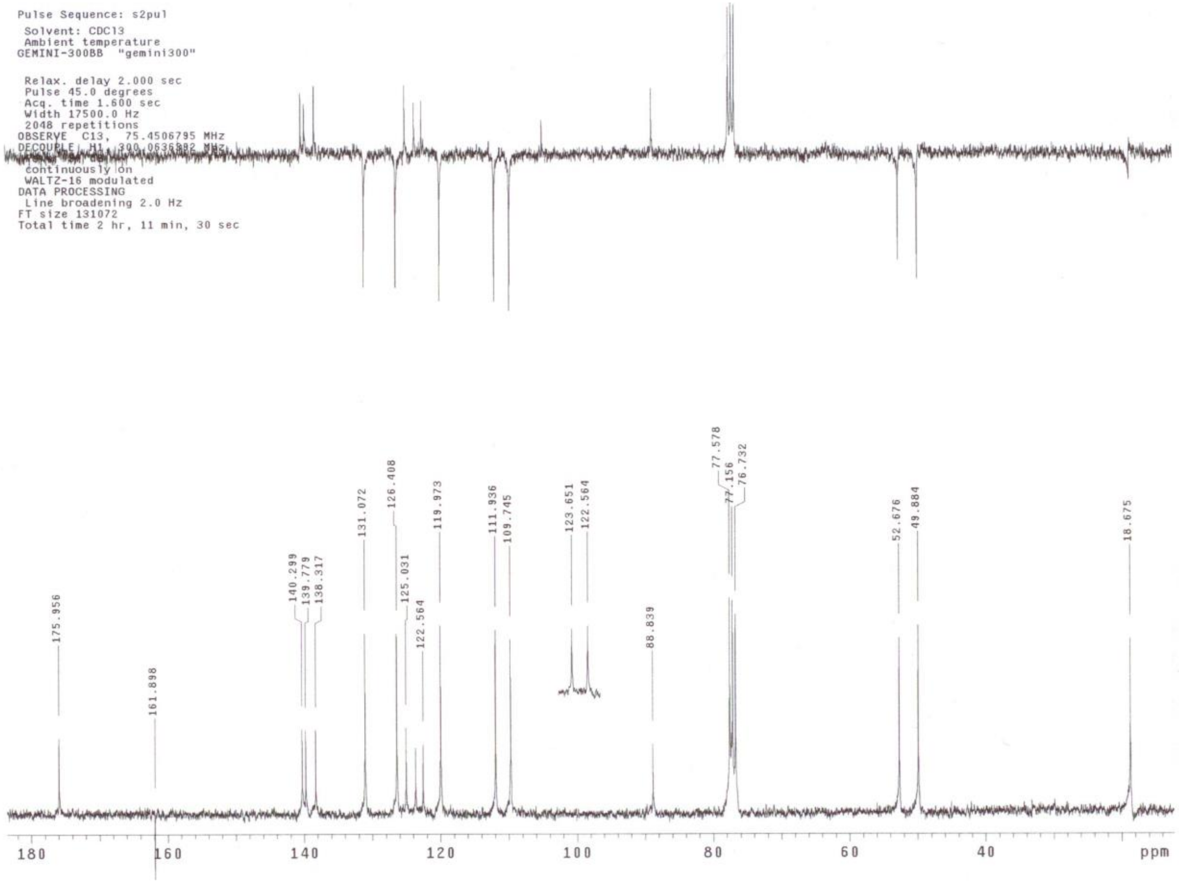

Figure S7b. The ^{13}C-NMR spectrum of Methyl 2-(3-iodo-6-chloro-9H-carbazol-2-yl)propanoate (**8**).

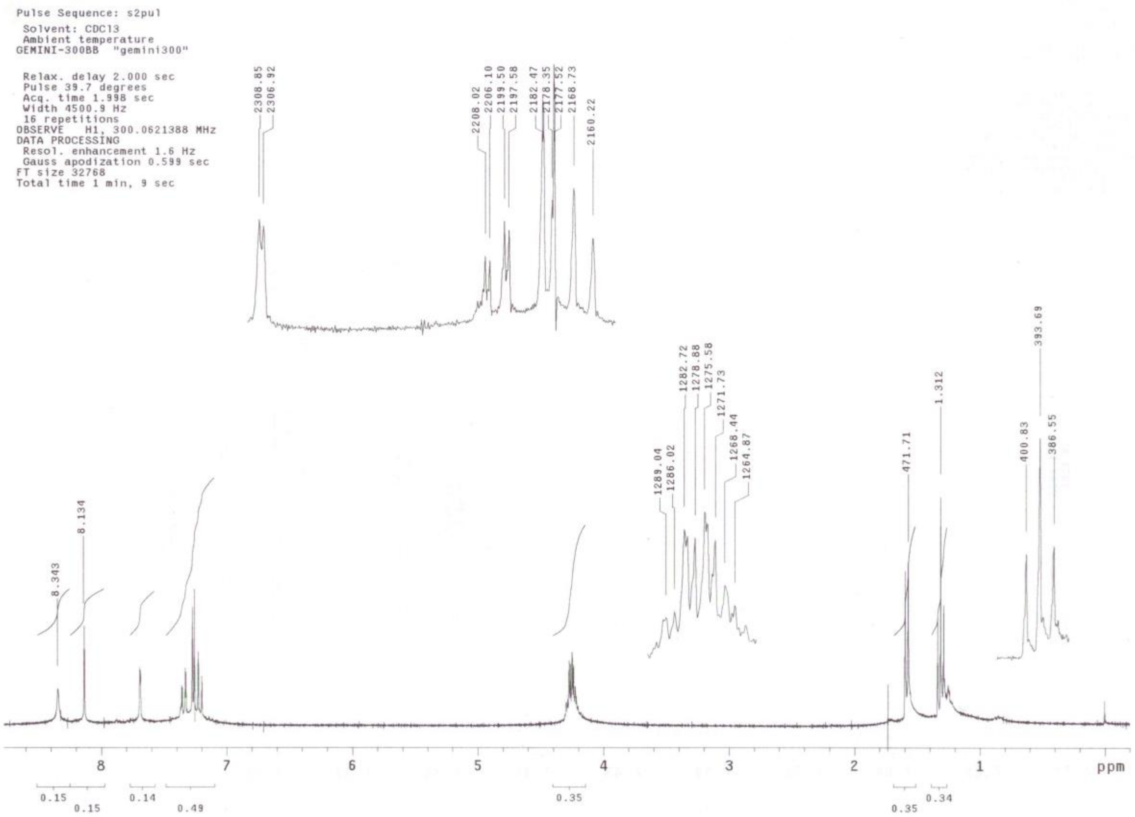

Figure S8a. The ^{1}H-NMR spectrum of Ethyl 2-(3-iodo-6-chloro-9H-carbazol-2-yl)propanoate (**9**).

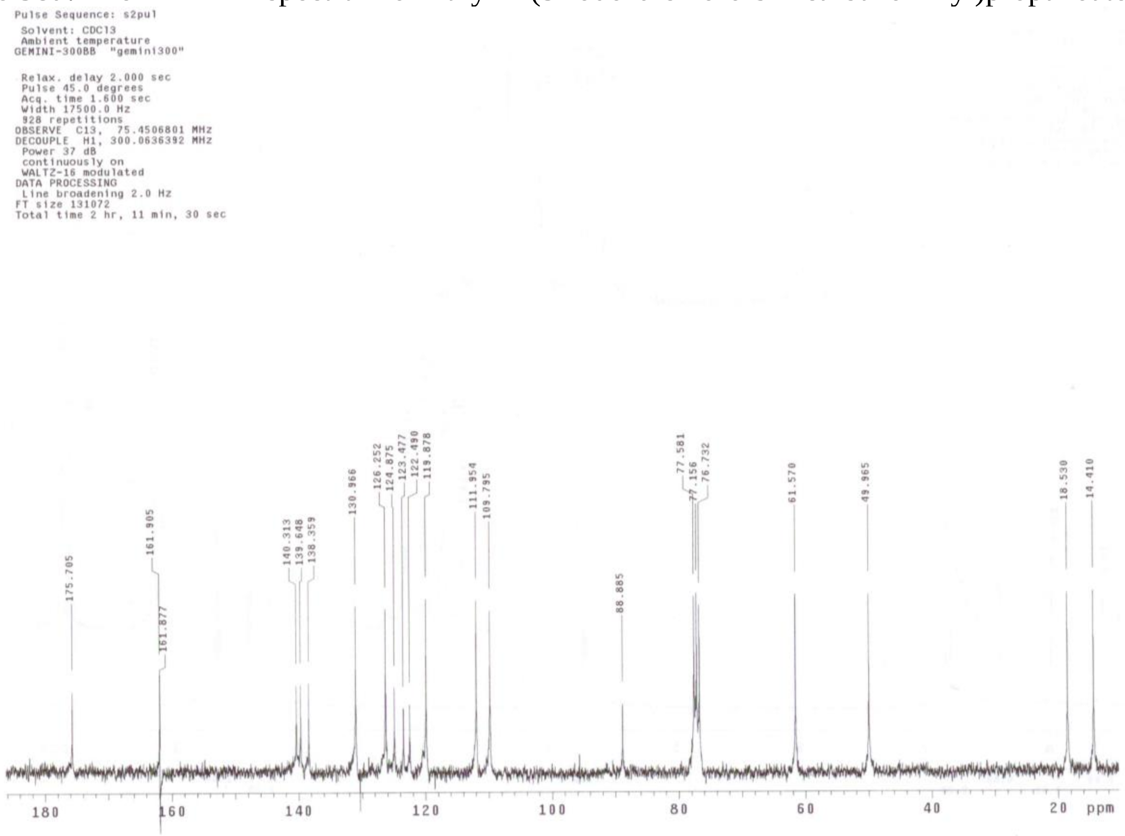

Figure S8b. The ^{13}C-NMR spectrum of Ethyl 2-(3-iodo-6-chloro-9H-carbazol-2-yl)propanoate (**9**).

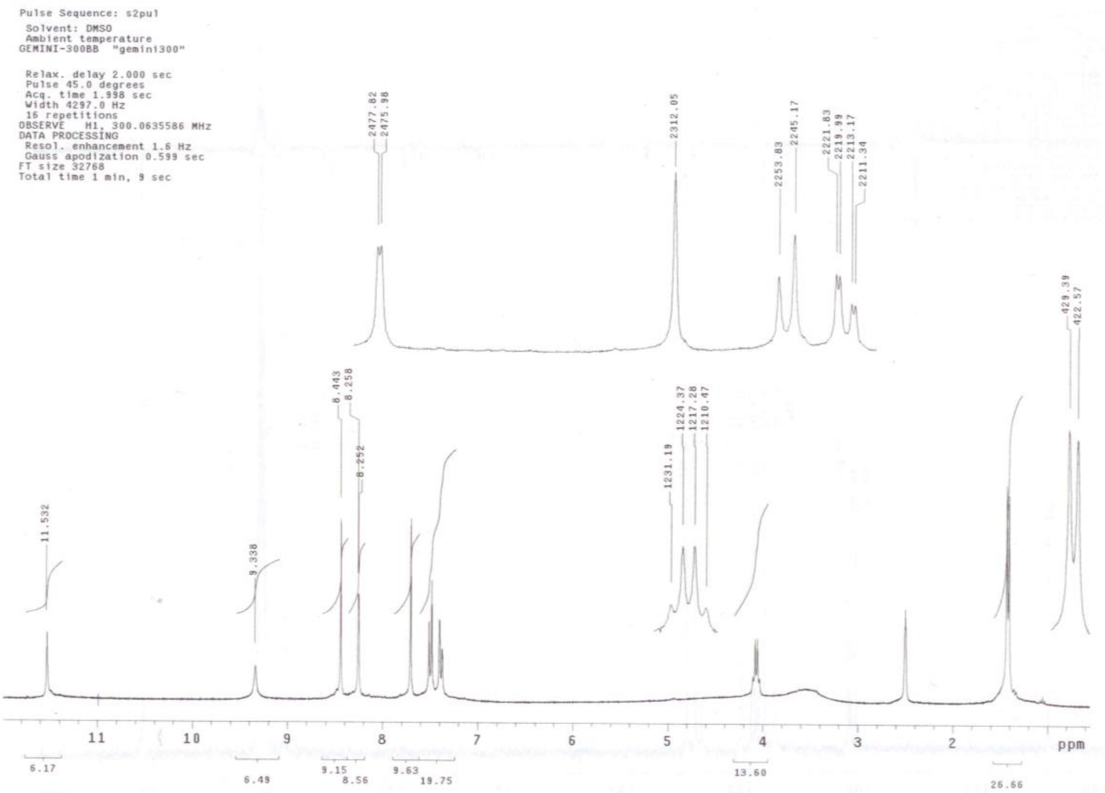

Figure S9a. The ^{1}H-NMR spectrum of Hydrazide of 2-(3-Bromo-6-chloro-9H-carbazol-2-yl)propanoic acid (**10**).

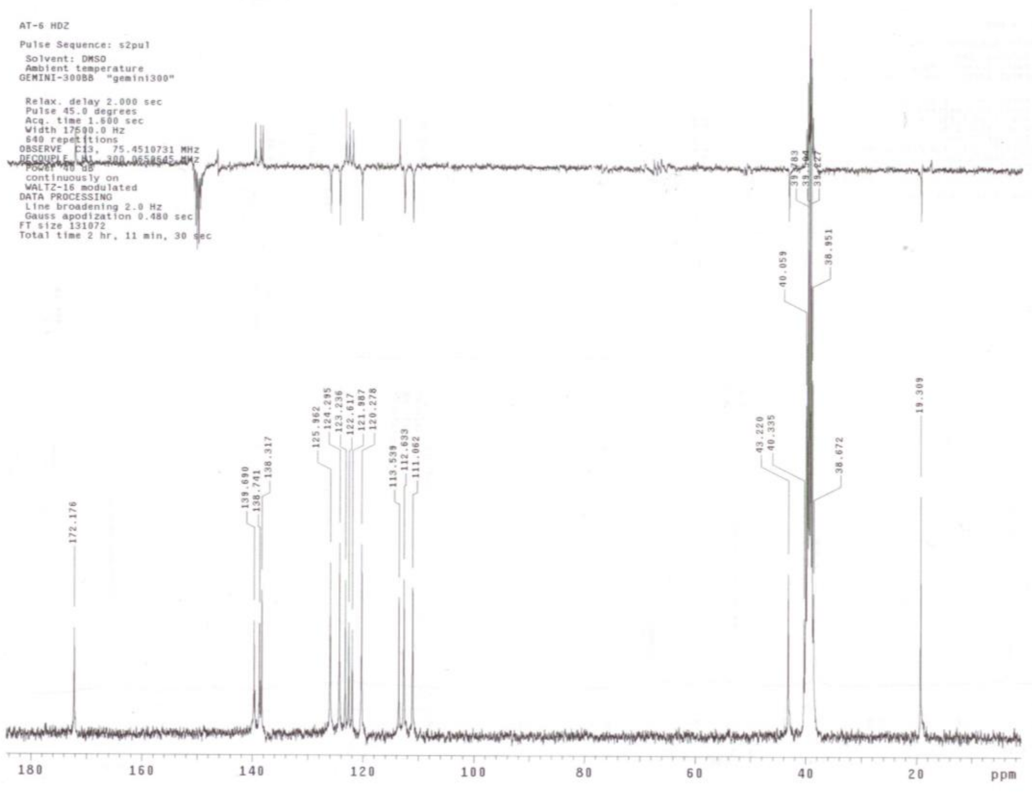

Figure S9b. The ^{1}H-NMR spectrum of Hydrazide of 2-(3-Bromo-6-chloro-9H-carbazol-2-yl)propanoic acid (**10**).

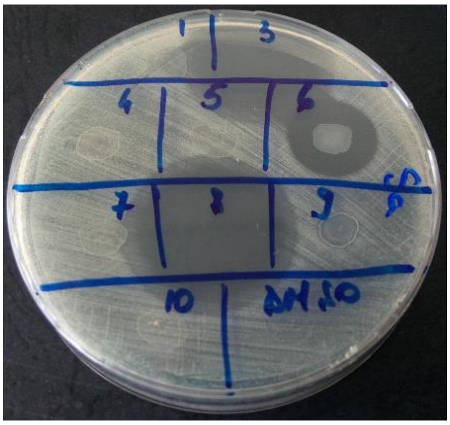

Staphylococcus aureus ATCC 25923

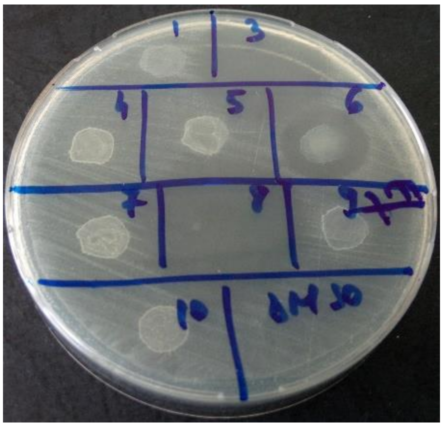

*Enterococcus faecalis*ATCC 29212

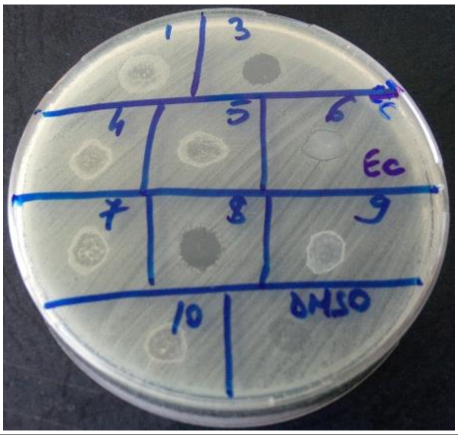

Escherichia coli ATCC 25922

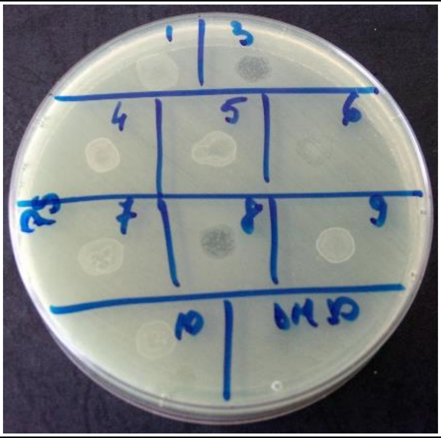

Pseudomonas aeruginosa ATCC 27853

Figure S10. The Qualitative screening of the antimicrobial activity of the tested compounds (1- compound **4**; 3- compound **2**; 4- compound **5**; 5- compound **7**; 6- compound **10**; 7- compound **6**; 8- compound **3**; 9- compound **8**; 10- compound **9**).
